# Information, ingestion, and impulsivity: The impact of technology-enabled healthy food labels on online grocery shopping in impulsive and non-impulsive consumers

**DOI:** 10.3389/fnut.2023.1129883

**Published:** 2023-03-28

**Authors:** Nikola Ljusic, Asle Fagerstrøm, Valdimar Sigurdsson, Erik Arntzen

**Affiliations:** ^1^Behavior and Technology Lab, School of Economics, Innovation, and Technology, Kristiania University College, Oslo, Norway; ^2^Centre for Research in Marketing and Consumer Psychology, Department of Business Administration, School of Social Sciences, Reykjavik University, Reykjavik, Iceland; ^3^Experimental Studies of Complex Human Behavior, Department of Behavioral Science, Faculty of Health Sciences, Oslo Metropolitan University, Oslo, Norway

**Keywords:** consumer behavior, technology, food labels, online grocery, delay discounting, impulsivity

## Abstract

**Introduction:**

Unhealthy food consumption is a problem for society, companies, and consumers. This study aims to contribute to knowledge regarding such issues by investigating how technology-enabled healthy food labels can impact food choice in an online grocery store context. We conceptualized unhealthy and healthy food choice as a matter of impulsivity problems. Three technology-enabled healthy food labels were derived based on variables that might impact self-control, and their influence on food choice was investigated.

**Methods:**

The empirical study consisted of three parts. In the first part, participants’ impulsivity was measured using an adjusting delay task. Part two investigated the effects of self-monitoring, pre-commitment, and social comparison-based technology-enabled healthy food labels on food choice in a hypothetical online grocery shopping setting using a choice-based conjoint experiment. Lastly, in the third part, three where demographical questions were asked.

**Results:**

The results (*n* = 405) show that self-monitoring, pre-commitment, and social comparison-based technology-enabled healthy food labels had the most to least impact on food choice in that order. Furthermore, the results indicate that self-monitoring and pre-commitment labels had more impact on the choice for impulsive compared to non-impulsive participants. Similarly, the results indicate that social comparison had more impact on choice for non-impulsive participants. These findings suggest that self-monitoring of previous healthy food choices might be more effective than pre-commitment based on discounts for healthy food products. However, these differences were minor.

**Discussion:**

This finding has managerial implications as grocery stores might increase their revenue by introducing self-monitoring labels in an online grocery shopping setting. Future research should investigate these technology-enabled healthy food labels in natural food purchase settings.

## 1. Introduction

Obesity is a problem worldwide. There is an increasing number of obese individuals across age, sex, geographical location, ethnicity, and socioeconomic status (1). There are now more obese than underweight individuals (2). It is associated with numerous diseases (3) and is a significant economic burden for society (4). Furthermore, a large body of evidence suggests that the food environment impacts obesity (5). As a result, the food industry is now receiving pressure from governments worldwide to decrease sales of unhealthy food products. This may lead to stricter government policies, such as introducing nutritional warning labels on food products if retailers, food manufacturers, and marketers do not adapt. In addition, it may limit consumers’ product options. In contrast to this hard strategy, companies may nudge consumers to purchase healthier options without restricting their food choices by altering the purchase situation (6). One proposed strategy for increasing healthier food choices is simplified front-of-package food labels (7) that signal how healthy a food product is. However, such labels do not always increase healthy food purchases, although such labels do help consumers identify which products are healthy (8, 9). Further, such labels may impact people that are obese differently than people who are not obese (10). Hence, identifying possibilities of new healthy food labels may be one way to increase healthy food purchases, and this has academic, managerial, and societal value.

Technology-enabled labels that present specific information may help consumers to commit to healthier food options over unhealthier food options. Specifically, they may be presented to increase healthy food purchases. These technology-enabled healthy food labels may provide personalized, dynamic, and real-time based information regarding the healthfulness of products in point-of-purchase situations (11). For instance, Shin et al. (12) investigated the effects of dynamic displays of technology-enabled labels on healthy food purchases in an online grocery store setting. They found that these labels were effective in increasing healthy food purchases. Furthermore, Fuchs et al. (13) investigated the effects of tailored food labels on self-reported intention to use and performance expectancy. Specifically, different scores regarding healthy foods were given depending on gender, age, physical activity levels, and body-mass index of participants. They found that such labels were perceived as more helpful, relevant, and recommendable than non-tailored healthy food labels.

One may present different technology-enabled healthy food labels to consumers based on their behavior, and one may present different labels for impulsive and non-impulsive consumers in an online grocery store context. Research shows that some behaviors are associated with obesity (14), and one of these behavioral predictors may be impulsivity (15). Impulsivity can be viewed as a *trans*-disease, as impulsive behaviors may lead to obesity, substance abuse, and other behavioral problems. As proposed by Foxall (16), in the context of impulsivity, consumer behavior may be on a continuum from routine to extreme consumer choice. Furthermore, Foxall (17) suggests that consumer behavior models that incorporate environmental factors may provide more predictive power compared to models that do not take these into consideration. Building on this, one may use choice experiments to identify environmental variables that may increase healthy food choice (18), and examine whether some environmental factors are more effective for increasing healthy food choice for impulsive and non-impulsive consumers than others. There exists some research suggesting that the purchase of food products in an online grocery store context results in healthier choices compared to offline grocery stores (19). However, this effect may occur due to delivery time, as consumers have to wait after making the order before receiving the products. This effect may not occur if the delivery time is made shorter if online grocers become more effective in reducing delivery time. Hence, online grocers may create technology-enabled healthy food labels that use variables that increase self-control to increase healthy food purchases and provide personalized technology-enabled healthy food labels for impulsive and non-impulsive consumers.

There exist several knowledge gaps in the literature related to the effects of healthy food labels. For instance, few research articles exist on technology-enabled healthy food labels and how they impact consumer behavior despite existing theoretical literature on incorporating psychological variables in food labeling (20). Furthermore, there exist studies that have investigated how impulsivity impacts the effects of food labels on consumer behavior (21–23). However, there is little research on this in an online grocery store setting. Most of these studies have used participants’ self-reported measurements of impulsivity rather than using choice behavior. Impulsivity measured by self-reports may produce different results than choice behavior (24). In addition, implementing technology-enabled healthy food labels may provide several benefits for companies, consumers, and society. For companies, such labels may create a competitive advantage by increasing healthy food sales, build brand equity, and generating positive word-of-mouth that may attract new customers. For consumers, it may increase health benefits and well-being. For society at large, it may reduce obesity rates and the concomitant economic burden. Hence, research regarding technology-enabled healthy food labels has significant societal and academic value. This paper thus aims to contribute to the body of knowledge by providing such research. The research questions of this study are as follows:

Research question 1: What is the relative impact of (a) self-monitoring-based, (b) pre-commitment-based, and (c) social comparison-based technology-enabled healthy food labels on choice behavior in a hypothetical grocery shopping setting?

Research question 2: How does the relative impact of these technology-enabled healthy food labels on choice behavior differ for impulsive and non-impulsive consumers?

The rest of the paper is structured as follows. First, a literature review and hypotheses for this paper are provided. Second, the methodology and results of this paper are presented. Third, findings and discussion are given. At last, implications and further research directions are explored.

Impulsivity may be measured by delay discounting. Delay discounting refers to the phenomenon where the value of a reward decreases as a function of increasing the delay to receive the reward (25). This relationship can be expressed by the hyperbolic formula presented in Equation 1 for delay (26):


$$V=\frac{A}{\left(1+k\,D\right)}$$

*V* is the subjective value of receiving a reward, *A* is the objective amount, *D* is the delay to receive the reward, and *k* is an empirically derived free parameter that determines the steepness of the subjective value. *A* higher *k* generates a steeper subjective value as a function of increasing delay than does a smaller *k* value. Typically, such functions are derived by asking individuals to make choices between receiving immediate and smaller or delayed and larger rewards, and then adjusting either the delay or amount. Participants’ indifference points between these two options are obtained and are used as a measure of empirical subjective value. Equation 1 has been shown to be more predictive of how the subjective value of a reward decreases as a function of delay than other models (e.g., traditional discounted utility model) and may describe preference reversals (27). Furthermore, some variables that moderate the effect of the probability of receiving a commodity on subjective value (probability discounting) may also be the same as variables that moderates the impact of the delay to receive a commodity on subjective value. However, evidence that these two constructs are the same phenomenon is small or moderates (28). In delay discounting, when the *k*-value is high, future events are discounted more than with lower *k*-values. Thus, impulsivity may be measured using *k*-values, as high k levels correspond to higher levels of impulsivity, while low k levels correspond to higher levels of non-impulsive (i.e., self-controlled) behaviors (for measurements of impulsivity see (29)).

High discounting rates are correlated with problematic health-related outcomes such as obesity and substance abuse (30), and discounting rewards depend on several factors. For instance, impulsivity may be due to genetic factors, as individuals who discount one commodity also tend to discount other commodities. However, it may also be influenced by current environmental factors. For instance, which type of reward is used (31, 32), cultural factors (33–35), and question framing (36, 37) may alter discounting rates. As exemplified by the Ainslie-Rachlin principle (38), there is a higher probability of choosing the immediate and smaller reward when the time between making a choice and receiving the reward is short. However, there is a higher probability of choosing the delayed and larger reward when both rewards are delayed by a constant. Using this knowledge, consumers may use external commitment devices to commit to choices that produce larger later rewards.

Delay and probability discounting have been used to investigate several factors influencing consumer behavior. For instance, it has been used to investigate the relationship between delivery fees and delay in e-commerce (39); rebates and for high and low-pricing products (40); online reviews and prices (41); hunger and discounting of food and non-food commodities (42). With regard to healthy food consumption, variables that may impact delay discounting may also impact healthy food choice (43). In accordance with this framework, there exists research that suggests that higher delay discounting of hypothetical momentary rewards is correlated with the purchase of unhealthy food products (44, 45) and that increasing delay for unhealthy foods may be used to increase the value of healthy food purchase (43).

Several systematic reviews and meta-analyses have identified that self-monitoring, pre-commitment, and social factors may increase non-impulsive behaviors (46–48). However, few studies have investigated how such strategies in the form of technology-enabled healthy food labels affect consumers at the point of purchase, and few have investigated their relative impact on choice behavior. For this study, the effects of technology-enabled healthy food labels that present self-monitoring of previous healthy food choice, pre-commitment options, and other consumers’ healthy food purchases on food choice behavior was investigated in point-of-purchase situations in a hypothetical online grocery store setting.

Self-monitoring refers to the recording and presentation of one’s own previous behavior to promote behavior change. Self-monitoring can function as a form of soft commitment (49). Specifically, observing one’s own previous patterns of choices may moderate the effects of long-term consequences on choice behavior without altering the immediate consequences of individual choices. Self-monitoring can be done actively, where individuals are required to record their behavior manually, or passively, where individuals may be presented with their own behavior history that is automatically recorded by a device. Research suggests that instructing individuals to actively record their choices may promote an increase in healthy food choices, and this has been investigated by using different technologies. For instance, Teasdale et al. (50) conducted a meta-analysis on remotely delivered strategies that used self-monitoring and tailored feedback and their effect on eating behavior. The strategies were delivered using paper reports, letters, booklets, and computers, and their results suggest that such strategies had a positive impact on eating behavior. Furthermore, Bartels et al. (51) conducted a systematic review of the effects of digital self-monitoring on improving health in middle-aged or older adults. The strategies were delivered using interactive voice response through using dials on telephones, personal digital assistants, short message services (SMS), smartphone apps, and computers. Their results show that most of the studies across behaviors lead to a change in at least one outcome measurement, including food and water consumption. Lim et al. (52) conducted a systematic review of the effects of technology apps to promote healthy food purchases and consumption. The devices that provided the strategies were mostly smartphones, and some used personal digital assistants. Their results show modest evidence for the efficacy of such strategies in improving healthy food purchase and consumption. These authors suggest that further research should explore passive automatic and personal feedback, that such digital health strategies could be incorporated into supermarket loyalty cards, and that real-time self-monitoring, feedback, and social incentives may increase healthy food choices. Hence, passive self-monitoring may be more effective in increasing the effects of long-term healthy food choices than active self-monitoring. One possible mechanism for this effect is that the presentation of previous higher values of non-impulsive behaviors may increase the probability of current non-impulsive behaviors. In addition, one may assume that non-impulsive individuals are more likely to be impacted by the presentation of their patterns of previously healthy food choice compared to impulsive individuals. This assumption is based on that non-impulsive behavior may be under the influence of temporally extended contingencies (49), such as environmental events that occur as a function of patterns of choices. Based on this, the following hypotheses are proposed:

H1: The presentation of food products in combination with higher values of prior healthy food choices for such products increases the probability of choosing of such products compared to their absence.

H2: The effects described in H1 will be greater for non-impulsive consumers than impulsive consumers.

Pre-commitment may refer to the voluntary act of changing the immediate consequences of individual choice to set the occasion for choosing larger-later rewards. Specifically, a commitment response that removes future available choices or that imposes a cost for certain choices (53, 54) in order to promote behavior change may be one way of defining pre-commitment. For instance, when consumers prefer healthy over unhealthy food when the time between making a choice and receiving the reward is large, then they can use hard commitment devices that provide additional consequences of their future individual choices. There exist studies that have investigated the effects of pre-ordering healthy food purchases and choice. For instance, Stites et al. (55) investigated the combined effects of pre-ordering lunch online, mindful eating training, fat information, and price reductions on healthy food purchases by employers in a hospital. Their results show that individuals allocated to the treatment condition purchased on average fewer calories and fat content and had a higher degree of mindful eating than individuals in the control condition. Similarly, Miller et al. (56) investigated the effects of pre-ordering compared to pre-ordering with a behavioral nudge. The nudge consisted of messages suggesting that all the components of a healthy meal or messages stating that the participants had selected a balanced meal if they selected all the healthy components. They found that participants in the pre-ordering condition had a higher average selection of fruit, vegetables, and milk products than individuals in the control condition. Furthermore, participants in the pre-ordering and behavioral nudge condition chose on average healthier products than the participants in the pre-ordering-only condition. Schwartz et al. (57) examined healthy food purchases as a function of pre-commitment by self-imposed aversive consequences. Specifically, households were enrolled in an incentive program that gave discounts on food products. The strategy consisted of an increase in the price of food products if they did not increase their prior healthy food purchase. Their results show that roughly one-third of the recruited households agreed to participate in the study. These households had higher healthy food purchases than the control group (and households that declined to participate). These studies suggest that pre-commitment may increase healthy food purchases. However, little research exists on the relationship between pre-commitment and the choice of healthy products for impulsive and non-impulsive consumers. In addition, one may assume that immediate environmental variables that may alter choice are more impactful for impulsive consumers than non-impulsive consumers. This assumption is based on that impulsive behavior may be under the influence of temporally narrow contingencies (49). Based on this, the following hypotheses are proposed:

H3: The presentation of food products in combination with pre-commitment to healthy food choice will increase the probability of choice for such products compared to the absence of pre-commitment.

H4: The effects described in H3 will be greater for impulsive consumers compared to non-impulsive consumers.

Social proof refers to the phenomenon whereby individuals tend to copy other people’s behavior when they are uncertain regarding what choices are correct in a given situation (58). Research has examined how social proof in the context of information sources, social identity, and self-control may impact healthy food choices. However, few articles have examined the effects of personalized healthfulness information on food basket choice when it is low or high compared to other consumers’ choices. Sigurdsson et al. (59) investigated the effects of different sources of social proof on the hypothetical choice and purchase of fresh fish. Specifically, the quality of the product was based on other consumers’ ratings by using a “Top Seller” label or authoritative sources by using a “Store’s Choice” label. Their first and second study found that other consumers’ ratings had more impact on choice behavior in hypothetical online grocery and brick-and-mortar store settings. Their third study found that both labels were effective in increasing sales of fresh fish and ground beef. Furthermore, Liu et al. (60) investigated the effectiveness of social norms on eating behavior as moderated by social identity. They found that social-proof messages regarding healthy foods were effective in increasing self-reports regarding healthy eating behavior for individuals who identified with the social group that the message referred to. Furthermore, Salmon et al. (61) investigated the effects of social proof on low-fat cheese purchases of consumers with high or low self-reported self-control. Their study induced high or low self-control by using an ego-depletion. Their results show that social proof increased the average percentage of low-fat cheese purchases consumers allocated to the ego-depletion task compared to controls. However, individuals who did not perform the ego-depletion task purchased, on average less low-fat cheese. The authors suggest that high self-control individuals may have purchased other healthy food products and that these results do not necessarily show a negative effect of purchase behavior for highly self-controlled consumers. However, these results have been produced by another similar study. Gonçalves et al. (62) investigated the effects of social proof on fruits and vegetables purchases of soft, medium, and hard buyers of fruits and vegetables. Their results show that social proof increases healthy food purchases for all consumers except hard buyers. These articles indicate that social comparison presented by other consumers’ purchases increases food choices in impulsive consumers. In addition, consumers who already purchase healthy food may be assumed to have higher self-control than individuals who do not. Based on this assumption, social comparison may be more effective for impulsive consumers and may not be effective for non-impulsive consumers. Based on these studies, the following hypotheses are proposed:

H5: The presentation of food products in combination with higher values of social comparison increases the probability of choice of such products compared to the absence of social comparison in impulsive consumers.

H6: The presentation of food products in combination with higher values of social comparison decreases the probability of choice of such products compared to the absence of social comparison in non-impulsive consumers.

## 2. Materials and methods

### 2.1. Participants

Four hundred and twenty-three participants were recruited by using the crowdsourcing platform Prolific. The sample that was selected by the service was a balanced sample of citizens in the United Kingdom. This sample size is considered appropriate for conjoint experiments (63, 64). The participants were invited to participate in a consumer choice study for £8 per hour with an estimation of 15 min to complete the study. They were required to read and sign an informed consent form regarding their rights as participants in an experiment before joining the experiment. They were told they could leave the experiment at any time during the study. This study has been assessed that to be in accordance with the Norwegian privacy legislation by The Norwegian Agency for Shared Services in Education and Research.

### 2.2. Setting, materials, and apparatus

The experiment was performed using several online and computer services. First, Prolific was used to recruit and administer the link to the experiment to the participants. Second, Sawtooth Software Lighthouse Studio 9.14.2 was used to record the participants’ choices, present the procedure, and conduct data analysis. Third, Excel, RStudio, and the ggplot2 package were used for data analysis and visual representation of the data. This study was first pre-tested with 102 participants and later a second test with 303 participants, resulting in a total of 405 participants.

### 2.3. Procedure

The procedure consisted of three parts which were presented in the following order. The first part consisted of a 5-trial adjusting delay task (65) and was used to measure the participants’ impulsivity. In the second part, the three technology-enabled healthy food labels were introduced and then a choice-based conjoint experiment (63) was used to assess their relative impact on choice behavior. The third part consisted of asking demographic questions. The study was pre-tested by using the Sawtooth Software random response simulation. The authors provided a link to the experiment using Sawtooth Software servers to each participant by using the Prolific platform.

#### 2.3.1. Adjusting delay task

Participants were required to read the following instructions: “*The study consists of three parts. The purpose of the first part is to examine your economic choices. You will be presented with several hypothetical scenarios that consist of two options each. Choose the option that you prefer by clicking on it. Press ‘Next’ to continue.”*

**FIGURE 1:**
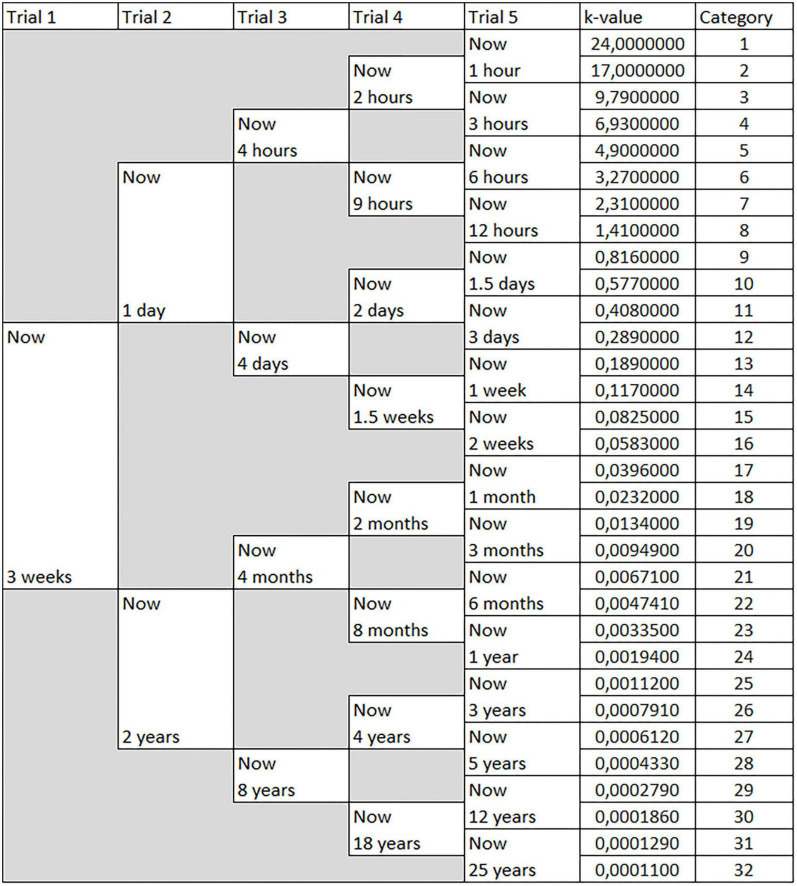
Overview of the adjusting delay task. This figure shows the hypothetical scenarios regarding the adjusting delay task. The trial number is indicated at the top. The initial delay during trial 1 was always three weeks. In trial 2, the participants were given the upper scenario if they selected now in trial 1 or were given the lower scenario if they selected three weeks in trial 1. The remaining trials had similar branching depending on the previous choice. K-values and the categories are specified on the right.

The 5-trial adjusting delay task consisted of presenting five trials each consisting of two hypothetical options. In all trials, participants were asked to choose between receiving hypothetical rewards of £50 now or receiving £100 in combination with a delay. The delayed reward was changed based on their previous choices. The delay in receiving the hypothetical reward in a trial was reduced if in the previous trial the participants chose to receive the reward now or increased if the participant chose to receive the reward later. The specific levels of delay in all trials are shown in Figure 1. The participants were required to choose one of two options before proceeding to the next trial. The participants could not go back to the previous trial once they submitted their answers, and the order of the options was randomized.

#### 2.3.2. Technology-enabled healthy food labels and choice-based conjoint experiment

After completing the adjusting delay task, participants were introduced to three technology-enabled healthy food labels and their relative effect on choice of food baskets in different hypothetical online grocery stores was examined. They were presented with the following introduction in part two: “*You have now finished the first part of the study, and you must now check off this box to confirm the end of part one. Part two will examine your preference regarding online grocery shopping. Press ‘Next’ to proceed.”*

They were later presented with the following instructions: “*Imagine that you are about to order a food basket by using an online grocery store. In these scenarios, you decide to compare three different online grocery stores before deciding which to choose. Each scenario has labels that will help you in the choice process.”* The participants were later presented with three technology-enabled healthy food labels successively. They were first presented with a symbol, then text that explained the symbol, and lastly, with a test that required them to match the symbol and the prior text.

For the introduction to the Streak label, the participants were shown an image of a blue square, and they were told that this was the healthy Streak label and instructed to press “next” to continue. Later, they were presented with the same image with the following text underneath. “*This label shows how many previous healthy orders in a row you have made. In this case, a healthy order is defined as having at least 50% of items in the basket that are labeled healthy by the Traffic Light Food Labelling System. If you choose this basket, you continue your healthy streak.”* They were required to press “Next” to continue during the presence of this text. Next, the participants were presented with the same square with three multiple-choice options. One of the options was the same text as during the introduction of the Streak label. Participants who selected this option were told they were correct and proceeded to the condition that presented the next label. Participants who selected either of the other two options were told that their answers were incorrect, redirected to the blue square, and the procedure was repeated.

For the introduction to the Incentive label, the participants were presented with an image of a white circle and they were told that this was the healthy Incentive label and instructed to press “Next” to continue. Later, the same image with the following text was presented: “*This label appears when you have a minimum of 30% fruits and vegetables in the basket. If you choose this option, you get a 10 % discount on this and your next purchase that also meets this requirement.”* Similarly, the participants were required to press “Next,” after which three multiple-choice options were presented. Likewise, participants were redirected to the label’s introduction if they selected options other than the original text. They continued to the next section if they selected the original text.

For the introduction to the Comparison label, the participants were presented with a pink triangle and were told that this was the healthy Comparison label and instructed to press “Next” to continue. Later, the same image with the following text underneath was presented: “*This label shows the percentage of groceries in your basket that are labeled healthy by the Traffic Light Food Labelling System ^®^ compared to what other consumers in your area have bought.” Similarly, three multiple-choice options were presented after selecting “Next”*. Likewise, participants were redirected to the label’s introduction if they selected options other than the original text. They continued to the next phase if they selected the original text. The text of the multiple-choice options is shown in Appendix A. All options were presented in random order.

**FIGURE 2:**
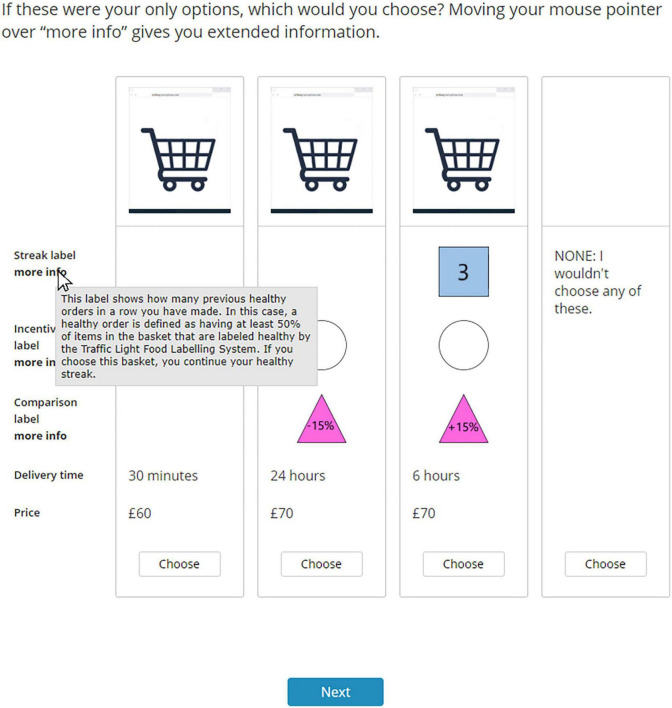
Example of a trial in the choice-based conjoint experiment. This figure shows an example of a choice trial in the choice-based conjoint experiment. The independent variables are on the right and the specific levels within each profile are indicated.

The participants were presented with a choice-based conjoint experiment right after the introduction to the labels. A conjoint experiment consists of a combination of generating experimental design and the usage of multivariate statistics to investigate the relative impact of multiple independent variables (63). Specifically, it consists of generating combinations of several values of independent variables, and their effect on decision-making is then evaluated. In a choice-based conjoint experiment, several profiles are presented and the participants are instructed to choose one among these profiles. In this study, the participants were presented with a choice-based conjoint experiment with several profiles within a trial, and their choices regarding these profiles were recorded. Each profile had information associated with it; this information was the independent variables in this study. This study used a full-profile method that presented all the independent variables simultaneously when a profile was presented. The choice trial consisted of three profiles and a “None” option where the latter was always positioned to the right. The participants had to select one of four options and press next to proceed to the “Next” trial. Each participant was presented with 12 choice trials, and the order of the trials was randomized to rule out order effects (66). A balanced overlap method was used to design the profiles (67). This method consists of generating choice trials where the profiles have combinations of values of independent variables that have low correlation. By using this method, the software (Lighthouse Studio 9.14.2) generated 300 different sets and each set had 12 choice trials. Each participant was presented with one of these 300 sets. The participants could access the information of each label provided in the instructions by hovering their cursor over the “more info” text underneath the names of the independent variables. An example of a trial is shown in Figure 2. The participants were presented with the following instruction before the choice-based conjoint experiment and between the 12 trials: “*You will now be presented with the 1st out of 12 different hypothetical purchase situations. These situations are independent of each other, and your choices in one situation do not impact the next. Thus, answer as you would have done in a real-life purchase situation.”* The instructions specified which trials were presented (i.e., 1st, 2nd, 3rd, … 12th).

##### 2.3.2.1. Independent variables

Five independent variables were used. Three of these were self-monitoring, pre-commitment, and social comparison based technology-enabled healthy food labels. Two additional independent variables were added to increase the realism of the choice experiment: delivery time and price.

First, the self-monitoring independent variable consisted of the following levels: “blank,” “square with number 2,” and “square with number 3.”

Second, the pre-commitment independent variable consisted of the following levels: “blank” and “circle.”

Third, the social comparison independent variable consisted of the following levels: “blank,” “triangle with −15%,” and “triangle with +15%.”

Fourth, the delivery time independent variable consisted of the following levels: “30 min,” “6 h,” and “24 h.” These levels were derived by examining the earliest delivery time options of five online grocery stores in London, England.

Fifth, the price independent variable consisted of the following levels: “£60,” “£70,” and “£80.” These levels were derived by examining the average amount spent per basket in English online grocery stores. These levels were set lower than the average amount spent per basket to decrease “None” option choices.

##### 2.3.2.2. Dependent variable

The dependent variable was choice behavior among profiles within a trial.

#### 2.3.3. Demographical questionnaire

After completing the choice-based conjoint experiment, participants were asked questions regarding their gender, age, household status, personal income last year, frequency of previous online shopping, product categories purchased online, frequency of purchasing food online, and food allergies.

### 2.4. Data analysis

Several data analysis methods were used. First, the frequency of participants across k-value categories was analyzed. Second, impulsive and non-impulsive individuals were classified by ranking them from high to low k-values according to the adjusting delay task. The half with the highest *k*-values were impulsive individuals, and the other half with the lowest *k*-values were defined as non-impulsive individuals. Three participant groups were formed, and these were based on (a) all participants, (b) impulsive participants’, and (c) non-impulsive participant. All of the groups’ data were used for statistical analyses. Second, logistic regression and Hierarchical Bayesian modeling based on aggregated data were used to estimate the impact of the independent variables and their levels on choice behavior. Logistic regression was employed by using maximum likelihood estimation for the main-effects of the relationship between binary choice behavior and the levels of the independent variables with five iterations. The regression coefficient for each level, standard error, and log-likelihood for the model was calculated. The importance score of the independent variables was calculated by taking the range of the regression coefficients of the levels within the independent variables and calculating the proportion of these values of one independent variable compared to the others. The impact of the independent variables for each participant was estimated using Hierarchical Bayesian modeling. This was done by estimating the impact of change at each level by using aggregate data of all participants and using such information to estimate the impact of each level for each participant. There were 20,000 iterations using this method, and the last 10,000 iterations were used for analysis. The average Hierarchical Bayes estimation for each level with standard deviation was estimated. Latent class analysis was performed by deriving two and three classes based on the results of the estimations. Finally, demographical data were provided for all three groups.

## 3. Results

Four hundred and twenty-three participants were invited to perform a study regarding consumer choice. Eighteen did not complete the survey, and their responses were removed from the analysis. The analysis was performed based on the remaining 405 participants in total. The average participant completed the study by in 526.47 s (8.77 min), with a range of 153–2,822 s (2.55–47.03 min), and a standard deviation of 290.77 (4.84 min).

**FIGURE 3:**
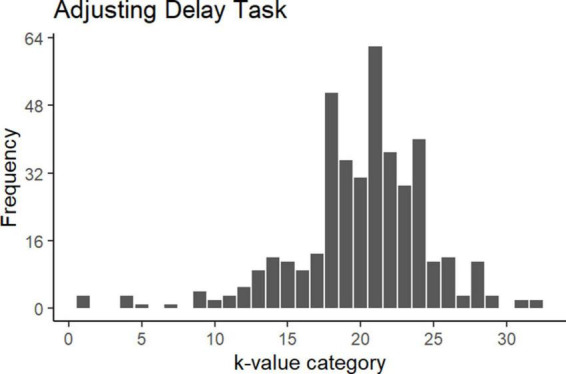
Results from adjusting delay task. This figure shows the frequency of each participant across the 32 different k-value categories. The frequency is indicated on the vertical axis and the k-value categories are indicated on the horizontal axis. Higher k-values are represented on the left, while lower k-values are represented on the right side of the graph.

The results from the adjusting delay task are shown in Figure 3. The figure shows that the category with the most participants was the 21st category (*k*-value = 0.0047), with a total of 62 participants. Based on these results, impulsive participants were defined as participants who completed the adjusting delay task and had a k-value of 24 to 0.0067 (from the 1st to the 20th category). Similarly, non-impulsive participants were defined as participants who completed the task and had a k-value of 0.0047–0.00011 (from the 21st to 32nd category.) As a result, 193 participants were classified as impulsive, and 212 were classified as non-impulsive.

The results of the demographic questions are shown in Table 1. Regarding all participants, the majority were males, and the most common age category was 25–34 years old. Most participants lived in a couple-household with children and had a personal annual income between £25,000 and £49,999. The majority shopped online once a week. Clothing and footwear were the most common items that were bought online, the majority of the participants bought groceries online at least once in a year, and the majority had no allergies. Regarding the impulsive participants, the majority were females, were between 25 and 34 years old, lived in a couple-household, had a personal annual income between £25,000 and £49,999, shopped online once every 2 weeks, bought online, majority of the participants bought groceries online at least once in a year, and had no allergies. Clothing and footwear were the most common type of products that were bought online. Regarding the non-impulsive participants, the majority were males, between 35 and 44 years old, lived in a couple-household, had a personal income between £25,000 and £49,999, and shopped online once a week. Books, music, movies, and games were the most common type of products bought online. Most participants bought groceries online at least once a year, and the majority had no allergies.

**TABLE 1:** The proportions of answers based on questions for all, impulsive, and non-impulsive participants.

Answers to the demographical questions			
	**All participants (*n* = 405)**	**Impulsive participants (*n* = 193)**	**Non-impulsive participants (*n* = 212)**
**1. What is your gender?**			
Male	50.12%	43.01%	56.60%
Female	49.63%	56.48%	43.40%
Non-binary/third gender	0.25%	0.52%	0.00%
Prefer not to say	0.00%	0.00%	0.00%
**2. What is your age?**			
18–24 years old	10.12%	10.88%	9.43%
25–34 years old	32.59%	35.75%	29.72%
35–44 years old	27.16%	23.83%	30.19%
45–54 years old	15.31%	16.58%	14.15%
55–64 years old	10.86%	9.33%	12.26%
65–74 years old	3.70%	3.11%	4.25%
75 years or older	0.25%	0.52%	0.00%
**3. What type of household do you belong to?**			
Couple household with children	40.99%	46.11%	36.32%
Couple household without children	29.63%	26.42%	32.55%
Single mother household	4.44%	5.18%	3.77%
Single father household	0.99%	0.52%	1.42%
Single person household	15.80%	13.99%	17.45%
Other	8.15%	7.77%	8.49%
**4. Which of these describes your personal income last year?**			
£0	0.99%	1.04%	0.94%
£1 to £9,999	12.84%	11.92%	13.68%
£10,000 to £24,999	29.63%	32.12%	27.36%
£25,000 to £49,999	39.01%	38.34%	39.62%
£50,000 to £74,999	9.63%	9.84%	9.43%
£75,000 to £99,999	0.74%	0.52%	0.94%
£100,000 or more	0.74%	0.00%	1.42%
Prefer not to answer	6.42%	0.62%	6.60%
**5. How often do you shop online?**			
Once a week	31.60%	29.02%	33.96%
Once every 2 weeks	26.42%	30.57%	22.64%
Once a month	19.26%	20.21%	18.40%
Around 3–4 times per quarter	12.35%	11.92%	12.74%
Once every 3 months	8.89%	7.77%	9.91%
I have not shopped online before	1.48%	0.52%	2.36%
**6. What type of products have you bought online? Multiple answers are possible.**			
Books, music, movies, and games	80.49%	77.20%	83.49%
Toys	50.62%	52.85%	48.58%
Consumer electronics and computers	72.10%	70.98%	73.11%
Sport equipment	39.01%	41.45%	36.79%
Health and beauty (cosmetics)	61.23%	64.77%	58.02%
Clothing and footwear	82.96%	83.94%	82.08%
Jewelry/watches	31.85%	33.16%	30.66%
Household appliances	65.43%	65.28%	65.57%
Do it yourself/home improvement	40.25%	36.27%	43.87%
Furniture and homeware	50.86%	51.30%	50.47%
Grocery	73.33%	75.13%	71.70%
None	0.49%	0.52%	0.47%
**7. How often do you purchase groceries online?**			
At least once in a year	35.56%	34.20%	36.79%
At least once in 6 months	20.74%	21.24%	20.28%
At least once in a month	26.91%	31.61%	22.64%
At least once a week	16.79%	12.95%	20.28%
**8. Do you have any allergies?**			
No	86.67%	84.97%	88.21%
Yes	13.33%	15.03%	11.79%

**FIGURE 4:**
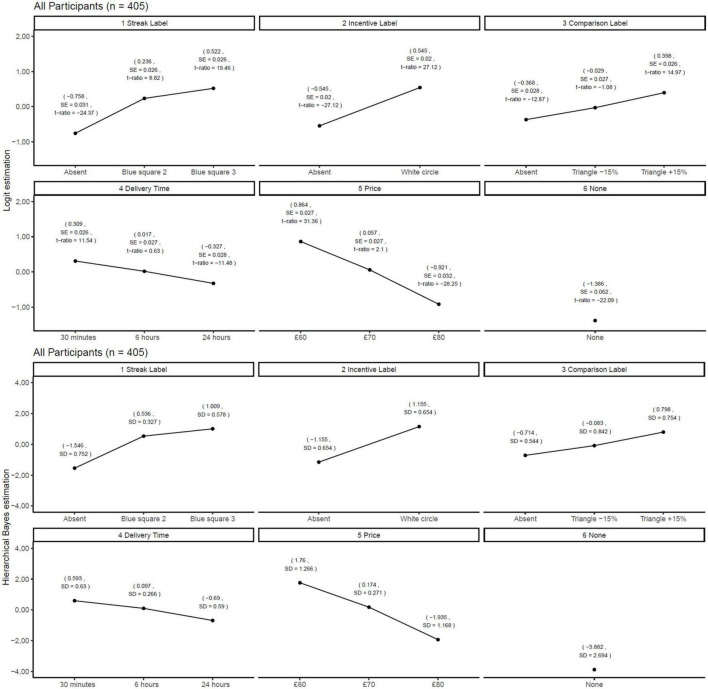
Results from estimated impact on choice on all participants. This figure shows the estimated impact of the independent variables on choice behavior. The name of the independent variables, their levels, the results of the logistic regression, and hierarchical Bayes from top to bottom.

**FIGURE 5:**
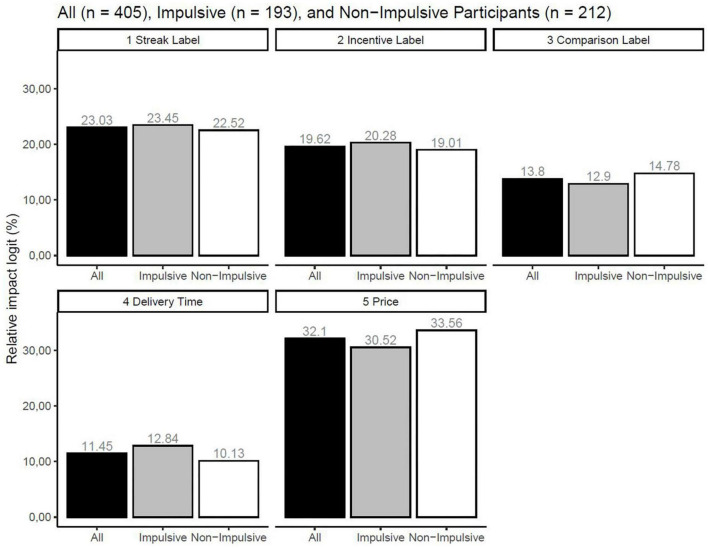
Relative impact of the independent variables for all, impulsive, and non-impulsive participants.

The results of the conjoint experiment based on all participants are shown in Figure 4. The results were the same when using logistic regression and Hierarchical Bayes estimation. Regarding the Streak label, the blue square with the number 3 was chosen more often than the blue square with the number 2, and the blue square with the number 2 was chosen more often than the absence of the Streak label. Regarding the Incentive label, the white circle was estimated to be chosen more often than the absence of Incentive labels. With regard to the Comparison label, the triangle with +15% was chosen more often than triangle with −15%, and the latter was chosen more often compared to the absence of the Comparison label. Regarding the delivery time, 30 min was chosen more often than 6 h, and the latter was chosen more often than 24 h. With regard to price, £60 was chosen more often than $70, and the latter was chosen more often than £80. The log-likelihood for the null model was −6,737.39, and the log-likelihood for the estimated model was −4,594.24, with a total difference of 2,143.15. In addition, the results from the logistic regression coefficients of the Comparison label based on impulsive participants were as follows: absent = −0.36, the triangle with −15% = −0.03, and the triangle with +15% = 0.39. The Hierarchical Bayes estimations for the same participants were as follows: absent = −0.70 (SD = 0.54), the triangle with −15% = −0.06 (SD = 0.91), and the triangle with +15% = 0.76 (SD = 0.74.) The logistic regression coefficients of the Comparison label based on non-impulsive participants were as follows: absent = −0.38, the triangle −15% = 0.03, and the triangle with +15% = 0.41. The Hierarchical Bayes estimations for the same participants were as follows: absent = −0.80 (SD = 0.67), the triangle with −15% = −0.11 (SD = 0.85), and the triangle with +15% = 0.92 (SD = 0.86.) The relative impact of the Streak label, Incentive label, Comparison label, delivery time, and price and Latent Class analyses based on these for all participants, impulsive participants, and non-impulsive participants are shown in Figure 5.

When comparing each group with itself, the results show a similar relative impact for all participants, including impulsive and non-impulsive participants. Specifically, price, Streak label, Incentive label, Comparison label, and delivery time had the most to least impact on choice in that order, using logistic regression and Hierarchical Bayes estimation. When comparing across the groups, the Streak label and incentive label had more impact on choice for impulsive participants than non-impulsive participants. Similarly, delivery time had more impact on impulsive participants compared to non-impulsive participants. In addition, price had less impact on choice for impulsive participants than non-impulsive participants. The log-likelihood for the null model based on impulsive participants was −3,210.66, and the log-likelihood model for the estimated model was −2,158.80, with a total difference of 1,051.85. The log-likelihood for the null model based on non-impulsive participants was −3,526,73, and the log-likelihood for the estimated model was −2,426.46, with a total difference of 1,100.26. When using three latent classes, the largest class shows that the Streak label and Incentive label had the most impact on choice, and the second largest shows that price and Incentive label had the most impact on choice for all participants, impulsive participants, and non-impulsive participants.

**FIGURE 6:**
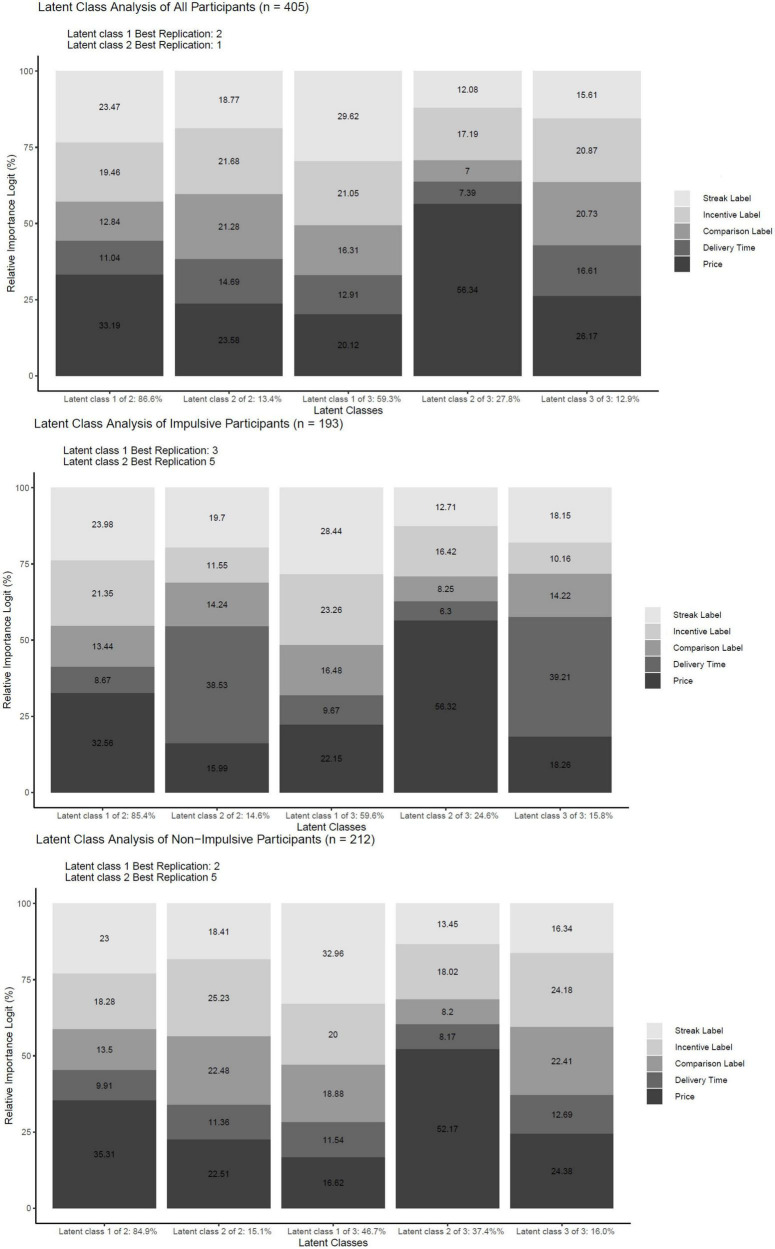
Results from latent class analysis for all, impulsive, and non-impulsive participants. This figure shows the results of the latent class analysis for all, impulsive, and non-impulsive participants.

The results presented here support H1, H3, and H5, while the they do not support H2, H4, and H6. Specifically, the results show that higher values of prior healthy food choice, pre-commitment to healthy foods, and higher social comparison increase the probability of choice behavior compared to the absence of these labels. Furthermore, the latent class analysis and relative impact of these three independent variables (presented in Figure 6) did not identify segments that differed with regard to impulsive and non-impulsive participants. When using logistic regression coefficients and Hierarchical Bayes estimations of the impact of the Comparison label, the results showed no negative impact of the triangle with +15% on choice behavior for non-impulsive participants.

## 4. Discussion

The main purpose of this study was to investigate whether choice behavior impacted by technology-enabled healthy food labels differed from impulsive and non-impulsive participants. Specifically, the relative impact of self-monitoring, pre-commitment and social comparison when presented as technology-enabled healthy food labels on choice behavior in a conjoint experiment was used. Impulsivity was measured through choice behavior by using an adjusting delay task.

This research contributes to two research fields. First, it relates to the emerging online grocery store and healthy food choice literature. Second, it relates to the general self-control literature and variables impacting healthy food choice. To the best of our knowledge, this is the first study to do so.

### 4.1. Internal validity

Overall, the results suggest that the self-monitoring, pre-commitment, and social comparison-based technology-enabled healthy food labels were the labels that had the most impact on choice behavior from most to least, in that order. In addition, the results indicate that self-monitoring and pre-commitment-based technology-enabled healthy food labels might be more effective for impulsive individuals than non-impulsive individuals. Furthermore, the results show that social comparison was more impactful on choice for non-impulsive participants than impulsive participants. However, clear segmentation based on latent class analysis regarding these results were not found, and definitive conclusions cannot be made based on these results.

With regard to self-monitoring-based technology-enabled healthy food labels, the results show that the presentation of higher values of prior healthy food choices increases choice behavior compared to its absence. Regarding pre-commitment-based technology-enabled healthy food labels, the findings show that the presence of pre-commitment to healthy food choice increases choice behavior compared to its absence. Furthermore, these results did not differ between impulsive and non-impulsive participants. With regard to social comparison-based technology-enabled healthy food labels, the results show that higher levels of social comparison increase choice behavior compared to its absence for impulsive participants. Lastly, the findings did not show that higher levels of social comparison decrease choice behavior compared to its absence for non-impulsive participants. In addition, the results from Figure 5 indicate that impulsive participants’ choices are more impacted by delivery time compared to non-impulsive participants and that non-impulsive participants are more price sensitive compared to impulsive participants. These results show some correspondence between the adjusting delay task and the choice-based conjoint experiment. Regarding the logit regression coefficients of the independent variables, all estimations had a standard error below 0.05 except for the “None” option. The highest standard error for the “None” option was observed for the impulsive participants, with a value of 0.09.

### 4.2. External validity

Consistent with prior research, this study identified segments of impulsive respondents whose choices were more impacted by delivery time compared to non-impulsive participants. In addition, the results in Table 1 show that impulsive and non-impulsive individuals have different preferences regarding what type of products are bought online. For instance, a higher proportion of non-impulsive participants stated that they bought products online that were in the category “Do it yourself/home improvement” than impulsive individuals. One possible explanation is that such products require more effort than other products. This can be related to previous research indicating that preference for some commodities is more impacted by the same variables that affect delay discounting.

With regard to self-monitoring of healthy food choice, the findings of this study are in accordance with articles that were used in the literature review, where self-monitoring may impact food and healthy choice. In addition, this study builds on previous calls to investigate the effects of automatic self-monitoring of previous food choice in a point-of-purchase situation which includes personal feedback. Moreover, this study also strengthens these findings by isolating the effects of self-monitoring of healthy food choice on food choice. Specifically, the results show that the presentation of higher values of healthy food choice alone can impact current food choice. Lastly, this study found that some of the effects of self-monitoring are generalizable to hypothetical online grocery shopping. With regard to pre-commitment to healthy food choice, the findings of this study support previous research in the sense that pre-commitment to healthy food choice might be an effective strategy for increasing healthy food choice. Specifically, price reductions might be effective in increasing fruit and vegetable choice, as indicated in the literature. Similarly, this effect was also observed in a hypothetical online grocery context. With regard to the social comparison of healthy food choice, the findings of this study show mixed support for previous research. This study found that positive social comparison increases food choice compared to its absence. However, the articles that were found in the literature review suggest that social comparison might have negative effects on food choice. For instance, Gonçalves et al. (62) found different effects of social comparison on food choice depending on whether the participants were frequent or non-frequent fruit and vegetable buyers. The findings in this study indicate that social comparison-based technology-enabled healthy food labels were more effective for non-impulsive participants. As indicated in Table 1, more non-impulsive participants stated that they bought groceries online at least once a week compared to impulsive consumers. The results presented in Figure 5, however, suggest that frequent fruit and vegetable buyers, in this case, non-impulsive participants, were more impacted by social comparison than impulsive participants. One possible interpretation is that such buyers are more sensitive to social comparison in an online grocery store context than in a physical store.

### 4.3. Implications and further research

There are several implications of these findings. First, the results show that consumers’ choices were more impacted by the Streak label than by Incentive labels. These finding that in some situations consumers prefer non-monetary over some discount monetary-based technology-enabled healthy food labels indicates that companies might use this technology to save costs while at the same time increase healthy food choice for consumers. Companies may use self-monitoring labels rather than providing a 10% discount on healthy foods to increase healthy food choice. Self-monitoring-based technology-enabled healthy food labels can benefit companies, consumers, and society at large. Second, developing these self-monitoring-based technology-enabled healthy food labels might not be expensive. Most online grocery stores require customers to create an account to purchase groceries. Online grocers can integrate this information into the customers’ accounts, which may be presented in point-of-purchase situations. Third, several considerations must be considered when implementing new technology. For instance, privacy, accurate data, ownership, and accessibility of data being collected must be considered (68). Fourth, the findings suggest that negative social comparison-based technology-enabled healthy food labels are preferred over the absence of such labels, indicating that the negative impact of these on food choice compared to their absence is not that detrimental for food choice. Fifth, implementing such technology-enabled healthy food labels might generate more engagement with the online grocery store, which may generate positive word-of-mouth. Lastly, not only can companies that implement these technology-enabled healthy food labels generate more revenue, but they can also provide higher consumer well-being by not restricting the consumers’ product options.

There are several considerations that future studies could investigate. First, these results might be specific to UK participants, and these results might depend on cultural factors as well. Second, what was considered healthy by the Streak label and Comparison-based labels were based on the Traffic Light Food Labelling System, a front-of-package food labeling system used in the UK. The Incentive label was, however, based on how many fruits and vegetables were in the hypothetical food basket. These differences may have impacted choice behavior. However, the Comparison label was the least impactful technology-enabled healthy food label in this study, and was based on the Traffic Light Food Labeling System. Third, some order effects might have affected choice behavior. Specifically, the order of the attributes was fixed in the choice experiment, which might be a confounding variable. In addition, the sequence of the introduction to the technology-enabled healthy food labels might also have impacted the results. Fourth, this study investigated hypothetical online grocery shopping and did not investigate the effects of these technology-enabled healthy food labels on actual purchases. The findings of this study may differ in a real online purchase situation. Lastly, the sample size of the latent class analysis of three groups might be too small to give robust findings, and they should be viewed as an indication. However, the logistic and Hierarchical Bayes estimations of the relative impact of the technology-enabled healthy food labels based on all participants, impulsive participants and non-impulsive participants, had an adequate sample size as indicated by the standard errors.

Several research topics should be investigated based on the findings of this study. First, future research should investigate how these technology-enabled healthy food labels impact actual purchases of healthy foods. Second, future research should also investigate the impact of other forms of technology-enabled healthy food labels on food choice. For instance, one might present technology-enabled healthy food labels that present the benefits of selecting healthy food baskets in terms of how one increases one’s life expectancy by selecting healthier options. Furthermore, one might highlight healthy foods not previously purchased at the point-of-purchase in an online grocery store to increase healthy food choice variety. In addition, many criteria exist for a healthy food product. One can ask what specific food products or categories are considered healthy for each consumer when creating an account for an online grocery store and highlight food products that are considered healthy for each consumer using technology-enabled healthy food labels. Third, this study investigated whether some technology-enabled healthy food labels were more effective for impulsive and non-impulsive consumers. Future findings may also investigate whether variables that impact probability discounting might impact healthy food choice. Specifically, some technology-enabled healthy food labels might be more effective for risky and risk-aversive consumers. As mentioned, unhealthy food consumption is associated with numerous diseases, and an increase in unhealthy food consumption increases the risk (or probability) of acquiring such diseases. Hence, variables that might impact risk-taking might be the same variables that impact healthy food choice.

## Data availability statement

The datasets presented in this article are not readily available because the data that has been used in this study is confidential. Requests to access the datasets should be directed to NL, nikola.kristiania@kristiania.no.

## Ethics statement

Ethical review and approval was not required for the study on human participants in accordance with the local legislation and institutional requirements. The patients/participants provided their written informed consent to participate in this study.

## Author contributions

NL came up with the idea and performed data analysis. NL and AF planned the study. VS and EA provided input on data presentation and refinement of concepts. All authors contributed to the discussion of the results and final manuscript.
