# Supplementary material for: Information, ingestion, and impulsivity: The impact of technology-enabled healthy food labels on online grocery shopping in impulsive and non-impulsive consumers
Source: Front Nutr. 2023 Mar 28;10:1129883. doi: 10.3389/fnut.2023.1129883 (PMC10099808; doi:10.3389/fnut.2023.1129883)
Supplement: Supplementary file 1 [file Table_1.DOCX]

Supplementary Material

# Supplementary Data

This is the healthy Streak label. What does it do?

Option 1 (correct option):
This label shows how many previous healthy orders in a row you have made. In this case, a healthy order is defined as having at least 50% of items in the basket that are labeled healthy by the Traffic Light Food Labelling System. If you choose this basket, you continue your healthy streak.

Option 2:
This label shows how many healthy food purchases you have done in a total of 3 weeks. It shows the percentage of items in my basket labeled green by the Traffic Light Food Labelling System ®.

Option 3:
This label shows how many food items in your current basket are considered healthy. It shows the percentage of items in my basket labeled healthy by the Traffic Light Food Labelling System ®.

This is the healthy Incentive label. What does it do?

Option 1 (correct option):
This label appears when you have a minimum of 30% fruits and vegetables in the basket. If you choose this option, you get a 10 % discount on this and your next purchase that also meets this requirement.

Option 2:
This label gives you the option to get newsletters on how to eat healthier. You can receive ads for items that are only labeled healthy by the Traffic Light Food Labelling System ®.

Option 3:
This label gives you the information regarding the salt content of the basket. It shows the percentage of items in my basket where the salt content was labeled unhealthy by the Traffic Light Food Labelling System ®.

This is the healthy Comparison label. What does it do?

Option 1 (correct option):
This label shows the percentage of groceries in your basket that are labeled healthy by the Traffic Light Food Labelling System ® compared to what other consumers in your area have bought.

Option 2:
This label provides an alternative basket that is a healthier option. The basket consists of a 10% increase in items that are labeled green by the Traffic Light Food Labelling System ®.

Option 3:
This label shows how healthy my current basket is based on the rating of other consumers. It shows the average rating done by other consumers that is based on a five-star rating.
